# Comparative evaluation of physics versus conventional forceps in tooth extraction

**DOI:** 10.6026/973206300222462

**Published:** 2026-04-30

**Authors:** Manish Sangtani, Geeti Vazdi Mitra, Bhumika Sangtani, Tejas Motiwale

**Affiliations:** 1 Department of Dentistry, District Hospital, Damoh, Madhya Pradesh, India; 2 Department of Dentistry, Sri Aurobindo University, Indore, Madhya Pradesh, India; 3 Department of Dentistry, City smile Dental Clinic, Damoh, Madhya Pradesh, India; 4 Department of Oral and Maxillofacial Surgery, Sri Aurobindo College of Dentistry, Indore, Madhya Pradesh, India

**Keywords:** Extraction forceps, physics forceps, non-carious teeth, carious teeth

## Abstract

Tooth extraction often results in trauma, leading to loss of alveolar bone and soft tissue. Therefore, it is of interest to compare the efficacy of physics forceps and conventional forceps in extracting non-carious and carious teeth. The results show significant differences in operating time, intra-operative complications and postoperative pain for carious teeth extractions, with physics forceps performing better. However, for non-carious teeth, only operating time was significantly reduce with physics forceps. Thus, we show the understanding of physics forceps as an effective tool for reducing complications and pain in carious tooth extractions.

## Background:

Extraction of tooth is the most common/routine surgical procedure to be performed in dental clinic. Most common reasons for extraction of tooth being caries in younger age group and periodontal problems in older age group [1]. Even today, extraction of tooth is the most dreaded dental procedure for the dentists as well as patients despite development of modern anaesthetic drugs & techniques [2]. However, exodontia is inherently traumatic procedure and some destruction and loss of surrounding hard and soft tissue is inevitable and dry socket is its most common complication [3]. This trauma results in loss of ridge dimensions which negatively affects aesthetic outcome post prosthetic rehabilitation and may preclude dental implant placement or result in food lodgement below fixed partial dentures [4]. Thus, atraumatic dental extraction techniques have gained prominence for preservation of gingival and bony architecture especially for immediate dental implant placement and one such technique is called Physics Forceps [5]. Powertome, Proximators, Periotomes and Benex Extractor, Sapian root removal system are other such instruments. Conventional dental forceps are actually 2 first-class levers connected with a hinge which acts as a fulcrum [6]. The force on the handles is magnified only to grasp the tooth and not to remove it from its socket and thus increased force does not provide any advantage in extraction and can rather fracture the tooth.

In contrast, Physics Forceps are based on physical principles of rotational force, torque and a lever and function as an elevator, rather than forceps [7]. The Physics Forceps (TM) is claimed to be an atraumatic extraction system that uses first class lever principle for simple & predictable removal of tooth from its socket and thus providing a positive patient experience [8]. Various studies have been conducted to compare the efficacy of physics forceps with claimed advantages of biomechanical principles applied and conventional forceps for simple extractions and for orthodontic extractions with mixed and equivocal results [9]. Therefore, it is of interest to assess the physics forceps with conventional forceps in 2 different situations i.e. simple extraction of non-carious teeth and carious decayed teeth.

## Materials and Methods:

This clinical study was conducted at the Department of Oral and Maxillofacial Surgery, Sri Aurobindo College of Dentistry, Indore, with 200 patients who required tooth extraction. After obtaining ethical clearance and informed consent, patients were divided into four groups: Group I (non-carious tooth extraction with physics forceps), Group II (non-carious tooth extraction with conventional forceps), Group III (carious tooth extraction with physics forceps) and Group IV (carious tooth extraction with conventional forceps). The armamentarium included Physics Forceps (GMX 100/200), conventional extraction forceps, Molt's no. 9 periosteal elevator and a set of instruments for upper and lower teeth extractions. Local anaesthesia was administered using 2% lignocaine with 1:200000 adrenalines. The procedure for extractions varied based on the group, with physics forceps requiring less force and rotational movements for non-carious and carious teeth, while conventional forceps used a more aggressive approach involving buccal and lingual tilts. Intra-operative complications such as gingival laceration, crown and root fractures and bone plate fracture were evaluated and postoperative pain was assessed on the first, third and seventh postoperative days using the Visual Analogue Scale (VAS). Data was collected using a prescribed proforma in the Master Chart, with intraoral peri-apical radiographs or Orthopantomograph for further assessment. Statistical analysis was conducted using descriptive statistics, T-tests and chi-square tests to evaluate the significance of differences between the groups, considering a p-value of less than 0.05 as statistically significant.

## Results:

In the non-carious (orthodontic) extraction group, 30 patients underwent 100 extractions, with 20 patients having 'all 4' symmetrical premolar extractions and the remaining undergoing either bilateral or unilateral premolar extractions. In the carious tooth extraction group, 88 patients underwent 100 extractions, with a male predominance (52 males and 36 females). The mean age of patients in the non- carious extraction group (Group 1 and Group 2) was similar, with Group 1 having a mean of 19.321 years and Group 2 having 19.286 years. In the carious extraction group, Group 3 had a mean age of 38.381 years and Group 4 had a mean age of 37.174 years, with no significant difference between the groups. Table 1 (see PDF) shows the tooth distribution in non-carious extractions, with a total of 100 extractions across upper and lower premolars. Table 2 (see PDF) displays the tooth distribution in carious extractions, including a variety of molars, premolars and incisors. The comparison of mean operating time between Group 1 and Group 2 (non-carious extractions) showed a significant difference, with Group 1 having a mean time of 34.460 seconds and Group 2 at 50.280 seconds (p<0.05). Similarly, Group 3 (carious extractions with physics forceps) had a significantly lower operating time of 81.600 seconds compared to Group 4 (153.460 seconds, p<0.05). In terms of complications, no significant differences were observed between the groups for gingival lacerations and bone plate fractures. However, crown fractures and root fractures were significantly higher in Group 4 (conventional forceps, carious extractions). In root fracture incidence, Group 4 had 12 cases compared to 4 in Group 3 (p<0.05). Table 3 (see PDF) presents the success scores, with Group 3 having significantly higher success rates compared to Group 4 for carious extractions. Table 4 (see PDF) displays post-operative pain scores, with no significant differences between Group 1 and Group 2. However, Group 4 (carious extractions with conventional forceps) had significantly higher pain scores at post-operative day 1 and day 3 compared to Group 3, with no significant difference at day 7. These findings suggest that physics forceps offer a more efficient and less traumatic option, particularly for carious tooth extractions, leading to reduced complications and postoperative pain.

Group (1 & 2): The association between Success Score and Group of patients which was found to be non-significant

Group (3 & 4): Success score in group 3 was significantly higher than group 4.

Group (1 & 2): The difference between the mean values of two groups was found to be statistically non-significant AT ALL POINTS.

Group (3 & 4): The mean VAS of 4th group is significantly higher than that of 3rd group at Day 1 and 3 both whereas no significant difference was found in mean pain score at day 7.

## Discussion:

The present study compared various parameters between orthodontic extractions using physics and conventional forceps. The mean age of patients in the non-carious extraction group was similar between the present study (19.3 years) and Patel *et al.* (2016) [10] (19.4 years). Significant differences were found in the operating times between physics and conventional forceps, with the physics forceps group showing significantly shorter times in both studies. The incidence of soft tissue trauma was low and not statistically significant in both groups, while no crown fractures were noted in the present study. Root fractures and bone plate fractures were minimal and not significant in most studies. Success scores showed no significant difference between the two groups in orthodontic extractions, though postoperative pain scores were similar at day 7. The present study demonstrates that physics forceps reduce operating time significantly compared to conventional forceps, especially in carious tooth extractions, with less postoperative pain and fewer complications such as crown and root fractures. In contrast, the study by Hariharan *et al.* (2014) [11] observed a longer operating time for both forceps types and did not find significant differences in complications such as gingival laceration, crown fractures, or root fractures between the two groups. In comparison with Kapila *et al.* (2020) [6], the present study also found significant differences in operating time between physics and conventional forceps, with physics forceps taking less time (81.6 seconds vs. 153.46 seconds in the present study and 252 seconds vs. 510 seconds. Both studies reported significantly lower postoperative pain in the physics forceps group, with the present study noting significant differences on day 1 and day 3, though no difference was found by day 7. These findings emphasize the advantages of physics forceps in terms of operating time, complications and postoperative pain reduction, particularly for carious tooth extractions. These results support the advantages of physics forceps in reducing operating time, postoperative pain and certain complications, especially in carious tooth extractions. However, further research, particularly long-term studies, is needed to assess the full benefits of physics forceps.

## Conclusion:

We show that physics forceps reduce operating time compared to conventional forceps, with fewer complications in teeth with grossly destroyed crowns. The use of physics forceps results in less trauma, leading to smoother post-operative healing and higher patient satisfaction. Despite its learning curve and higher cost, physics forceps have the potential to become a widely used tool in dental practice, especially for atraumatic extractions required for advanced prosthetic rehabilitation.
